# 806 Prevalence and Predictors of Stigma in the Burn Population

**DOI:** 10.1093/jbcr/iraf019.337

**Published:** 2025-04-01

**Authors:** Renee Noordzij, Ayumi Saito, Edward Santos, Caitlin Orton, Lewis Kazis, Colleen Ryan, Jeffrey Schneider

**Affiliations:** Spaulding Rehabilitation Hospital, Harvard Medical School; University of Washington; Spaulding Rehabilitation Hospital, Harvard Medical School; University of Washington; Boston University; Shriners Children’s - Boston and Massachusetts General Hospital; Spaulding Rehabilitation Hospital, Harvard Medical School

## Abstract

**Introduction:**

Stigma is defined as when someone is treated negatively or devalued due to a particular characteristic or attribute. People with physical appearances that differ from the expected, such as individuals with burn injury, are at risk for stigmatization. This may influence quality of life and can lead to social isolation. Therefore, this study aims to examine stigma and its predictors in the burn population.

**Methods:**

Adult burn survivors from a multicenter longitudinal database from 2015-24 were examined. The primary outcome variable was the self-reported Neuro-QoL stigma scale assessed at 6, 12, 24, and 60 months after injury. Overall scale and individual item scores were calculated at all time points. Linear mixed-effects models with random effects assessed demographic and clinical variables associated with stigma levels over time (overall scale and individual items with separate models).

**Results:**

A total of 787 adult burn survivors were included in the study. Most aspects of stigma, including overall stigma, improved over time. In contrast, item level data demonstrated more frequent reports of people avoiding looking at them over time (1.44 at 6 months to 1.55 at 60 months, SD=0.9 and 1.0, respectively). At 6 months post-injury, burn survivors’ mean stigma score was 49.3 (SD=8.7), which is near the general population norm of 50 and demonstrates that slightly over 50% of burn survivors are below population-based norms. Younger age, non-White race, larger burn size and substance use were all associated with higher levels of stigma (p< 0.05). Individual item data shows that amputation is associated with less unhappiness with appearance (p=0.008). Negative predictors of various individual items of the stigma scale included self-inflicted cause of injury, counseling services received, and anxiety medication use (p< 0.05).

**Conclusions:**

This study demonstrates that stigma is common and largely improves over time after burn injury. In addition, age, race, burn size, and substance use are predictors of overall stigma. Various demographic and clinical factors may also predict individual aspects of stigma, demonstrating the complexity of stigma and its application to the burn population.

**Applicability of Research to Practice:**

Understanding the prevalence and predictors of stigma can help target early interventions for those at high risk for its adverse effects in the burn population.

**Funding for the Study:**

NIDILRR #90DPBU0008; #90DPGE0004. Partial Support was obtained from Shriners Hospitals for Children (Grant #79136-BOS-23 and 79138-BOS-23).

